# Genetic Ablation of Bcl-x Attenuates Invasiveness without Affecting Apoptosis or Tumor Growth in a Mouse Model of Pancreatic Neuroendocrine Cancer

**DOI:** 10.1371/journal.pone.0004455

**Published:** 2009-02-11

**Authors:** Jeffrey H. Hager, Danielle B. Ulanet, Lothar Hennighausen, Douglas Hanahan

**Affiliations:** 1 Diabetes and Hellen Diller Family Comprehensive Cancer Centers, Department of Biochemistry & Biophysics, University of California San Francisco, San Francisco, California, United States of America; 2 Laboratory of Genetics and Physiology, The National Institute of Diabetes and Digestive and Kidney Diseases (NIDDK), National Institutes of Health, Bethesda, Maryland, United States of America; Deutsches Krebsforschungszentrum, Germany

## Abstract

Tumor cell death is modulated by an intrinsic cell death pathway controlled by the pro- and anti-apoptotic members of the Bcl-2 family. Up-regulation of anti-apoptotic Bcl-2 family members has been shown to suppress cell death in pre-clinical models of human cancer and is implicated in human tumor progression. Previous gain-of-function studies in the RIP1-Tag2 model of pancreatic islet carcinogenesis, involving uniform or focal/temporal over-expression of Bcl-x_L_, demonstrated accelerated tumor formation and growth. To specifically assess the role of endogenous Bcl-x in regulating apoptosis and tumor progression in this model, we engineered a pancreatic β-cell-specific knockout of both alleles of *Bcl-x* using the Cre-LoxP system of homologous recombination. Surprisingly, there was no appreciable effect on tumor cell apoptosis rates or on tumor growth in the *Bcl-x* knockout mice. Other anti-apoptotic Bcl-2 family members were expressed but not substantively altered at the mRNA level in the *Bcl-x*-null tumors, suggestive of redundancy without compensatory transcriptional up-regulation. Interestingly, the incidence of invasive carcinomas was reduced, and tumor cells lacking Bcl-x were impaired in invasion in a two-chamber trans-well assay under conditions mimicking hypoxia. Thus, while the function of Bcl-x in suppressing apoptosis and thereby promoting tumor growth is evidently redundant, genetic ablation implicates Bcl-x in selectively facilitating invasion, consistent with a recent report documenting a pro-invasive capability of Bcl-x_L_ upon exogenous over-expression.

## Introduction

For all cancerous lesions to increase in mass and malignant potential, cell proliferation must outstrip cell death [1]. To this end, human tumors engage or disengage a variety of pathways to escape or minimize apoptosis. The necessity to down-modulate apoptosis also stems from the fact that activating mutations in oncogenes, as well as overexpression of wild-type oncogene alleles, evoke not only hyperproliferation, but also cell death [2]–[4]. A considerable body of evidence supports the proposition that, without some degree of suppression of apoptosis, oncogene-expressing cells cannot progress past relatively small, pre-malignant proliferative lesions [1], [5].

The first direct genetic connection between modulation of apoptosis and tumor growth came from the discovery that expression of the *Bcl-2* gene was dysregulated via chromosomal translocation in follicular lymphoma, and that this gene is homologous to the *C. elegans* regulator of cell death, ced-9 [6]–[9]. Bcl-2 proved to be the prototype of a family of structurally related apoptotic regulators, divided into two sub-groups that alternatively suppress or induce apoptosis [10]. The anti-apoptotic arm of the Bcl-2 family includes Bcl-2, Bcl-x (multiple anti-apoptotic splice variants), Bcl-w, A1, Mcl-1 and Boo [10]–[12]. Activation of apoptosis in response to oncogene-induced hyperproliferation has been described in a variety of genetically engineered mouse models of cancer, and in particular in two models of pancreatic neuroendocrine islet cell cancer. In one such model, RIP1-Tag2 [13], [14], SV40 T-antigens are constitutively expressed in the β-cells of the endocrine pancreas under control of the rat insulin promoter, resulting in a multistage pathway to invasive carcinoma that is stochastic in nature and temporally protracted. At 2–3 weeks of age, the normal post-natal developmental proliferation of islet cells tapers off, and in adult mice there is a relatively slow turnover of β-cells, with a low proliferation rate offset by a low apoptotic rate, which results in a very modest increase in β-cell mass with age. In contrast, the β-cells of RIP1-Tag2 islets begin hyperproliferating at 3–4 weeks of age along with a concomitant activation of apoptosis. An early study reported that apoptosis peaked at the angiogenic islet stage but was significantly down-modulated in tumors, suggesting that resistance to cell death was a critical step of tumor development from angiogenic islet precursors [15]. A more recent study of this model has shown that down-modulation is not always the case, in that late-stage tumors (including invasive carcinomas) in the current backcross generations typically have apoptotic rates similar to those of angiogenic islets, suggesting a more subtle phenomena at play in expansive tumor growth (ref [16]). Two well-defined suppressors of apoptosis have been identified in this model: functional inactivation of p53 via large T-antigen expression, and up-regulation of the survival factor IGF-II [17], [18]. Genetic studies involving crosses to an IGF-II null allele indicated that it largely functions as a survival factor in this model, in that absence of IGF-II evoked much increased (5×) apoptotic rates in hyper-proliferative lesions, and the small tumors arising had a less malignant cellular phenotype; notably, the proliferation rate remained unchanged [18], [19].

A demonstration that anti-apoptotic members of the Bcl-2 family can modulate cell-death in this model came from experimental over-expression of *Bcl-x_L_* in β-cells via the rat insulin promoter (*RIP-Bcl-x_L_*), which resulted in suppressed apoptosis and an increase in tumor burden, along with a general “acceleration” of the tumor pathway [15]. More recently, the same RIP-*Bcl-x_L_* transgene has also been shown to suppress apoptosis in another mouse model of islet cell carcinoma, in which a myc fusion protein, myc^TAM^, was expressed in the β-cells under the control of the rat insulin promoter [20]. Myc^TAM^ is only active in the presence of tamoxifen, allowing for inducible and reversible gene expression. Postnatal treatment of mice with tamoxifen results in a rapid induction of myc^TAM^ expression concomitant with hyper-proliferation of the β-cells. Interestingly, this hyper-proliferation was accompanied by massive β-cell apoptosis that resulted in islet involution within 6–10 days. Over-expression of *Bcl-x_L_* via the same *RIP-Bcl-x_L_* transgene suppressed this apoptosis, resulting in an increase in β-cell mass and a rapid progression to invasive carcinomas. Thus, in two different transgenic mouse models of islet cell carcinoma, elicited by two functionally distinct oncoproteins, over-expression of *Bcl-x_L_* suppresses apoptosis, resulting in accelerated tumor formation. The results indicate that the apoptosis observed in these tumor types is, at least in part, mediated by the mitochondrial death pathway. In this report, we explore the role that endogenous Bcl-x plays in modulating apoptosis and tumor growth in the RIP1-Tag2 model via targeted deletion of the gene in the oncogene-expressing β-cells using the Cre/loxP system.

## Results

### Bcl-x expression in RIP1-Tag2 islet cell carcinogenesis

**Figure 1:**
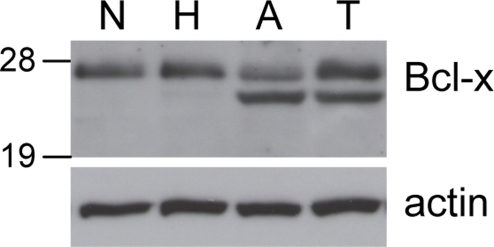
Bcl-x expression during RIP1-Tag2 tumor progression. Protein extracts from pools of normal, non-transgenic islets (N), hyperplastic islets (H), angiogenic islets (A), and tumors (T) from RIP1-Tag2 mice were immunoblotted with antibodies to Bcl-x and β-actin as loading control. The 27 kDa band represents Bcl-x_L_; the faster migrating band present in angiogenic islet and tumor extracts may represent the alternative splice variant, Bcl-x_β_.

In situ hybridization with a pan-isoform probe to pancreatic tissue sections from RIP1-Tag2 mice previously suggested that *Bcl-x* mRNA was modestly up-regulated in tumors relative to precursor lesions [15]. To confirm that Bcl-x is appreciably expressed at the protein level during islet tumorigenesis, we went on to assess the levels of Bcl-x protein by Western blotting using lysates from normal islets and from the discrete stages of pancreatic islet tumorigenesis in RIP1-Tag2 mice. As indicated in Figure 1, a band migrating at 27 kDa consistent with the Bcl-x_L_ isoform was detected at similar levels in normal islets and in the three neoplastic stages. In addition, a slightly faster migrating band, potentially corresponding to the alternative splice variant Bcl-x_β_ [21], [22], was detected in the angiogenic islet and tumor stage but not normal islets or the hyperplastic islet stage. Thus the overall levels of Bcl-x protein are modestly elevated in the angiogenic islet and tumor stages.

### Generating a β-cell specific gene knockout of bcl-x

**Figure 2:**
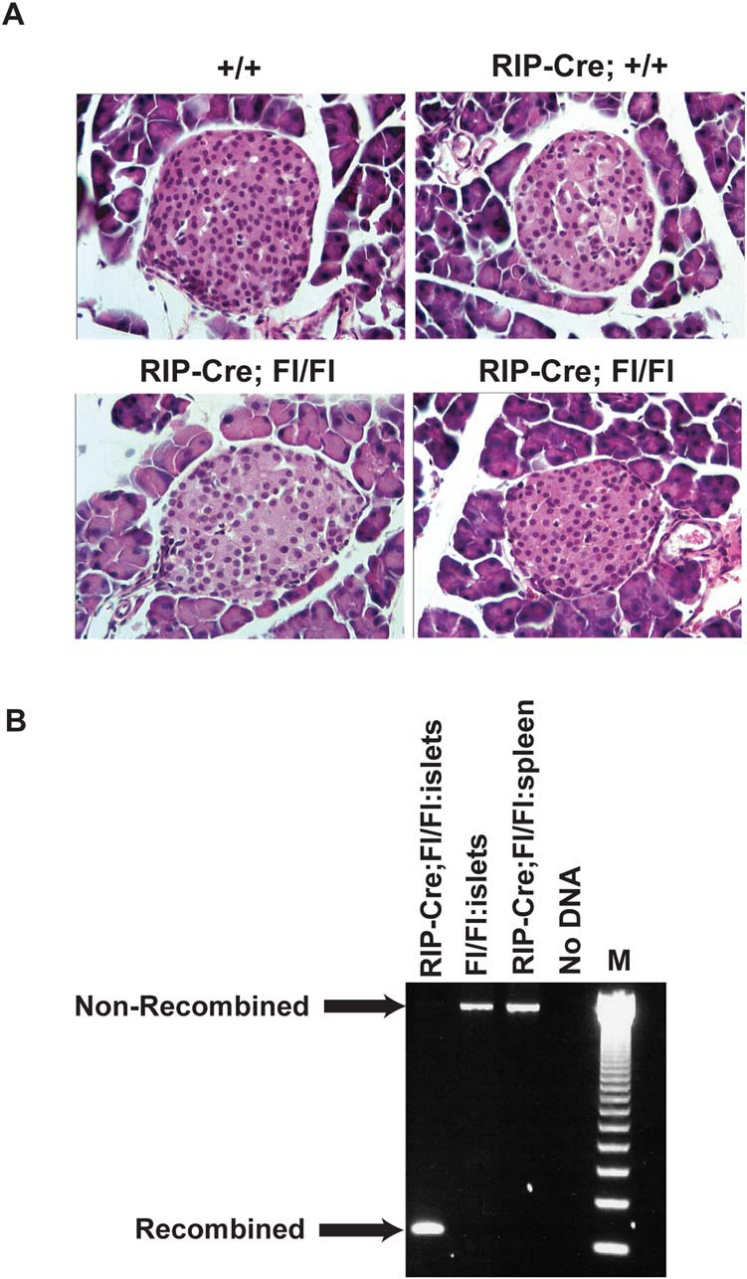
*RIP-Cre Bclx^fl/fl^* mice exhibit normal islet morphology and efficient *Bcl-x* recombination. (A) H&E stained pancreatic sections from 12 week old mice of the following genotypes: *Bcl-x^+/+^*; *RIP-Cre;Bcl-x^+/+^*; *and RIP-Cre;Bcl-x^fl/fll^*. Multiple sections of each of the animals depicted in photomicrographs were examined. (B) Genomic DNA from pools of pancreatic islets was isolated from mice of distinct genotypes. PCR was carried out using a primer that binds upstream of the 5′ loxP site and another primer that binds 3′ to the downstream loxP site. The PCR fragment of the non-recombined allele and the recombined allele are 2.9 kb and 150 bp, respectively. Pancreatic islets were isolated (Materials and Methods) from 4–6 animals of each genotype and pooled.

To assess the functional role of Bcl-x in RIP1-Tag2 tumorigenesis, we inactivated the *Bcl-x* gene in the β-cells of the endocrine pancreas using the Cre/loxP system of homologous recombination. The RIP-Cre line employed expresses Cre in approximately 82% of the β-cells, mediating efficient recombination at loxP sites [23], [24]. The “floxed” allele of *Bcl-x*, *Bcl-x^fl/fl^*, in which exons 1 and 2 are flanked by loxP sites, has been shown to be an efficient substrate for the Cre recombinase, producing a null allele following recombination in other tissues [23]–[25]. We first generated *RIP-Cre*, *Bcl-x^fl/fl^* mice (and various control genotypes) lacking the oncogene, and aged them out to >12 wks to assess possible effects on β cell development and homeostasis. *RIP-Cre*; *Bcl-x^fl/fl^* and *Bcl-x^fl/fl^* mice were born in expected Mendelian ratios, and reached adulthood without early lethality or overt pathology. We examined H&E stained pancreatic sections from *RIP-Cre:Bcl-x^fl/fl^*, and *Cre*-negative control mice using light microscopy. No obvious difference in islet size, distribution, or number was observed in a double-blind examination of these sections (Figure 2A). The results imply that β-cell development and islet morphogenesis were substantively normal in the absence of Bcl-x function, although subtle physiological effects cannot be excluded by this analysis. An alternative explanation for the lack of overt phenotype in *RIP-Cre*; *Bcl-x^fl/fl^* mice would be that the β-cells with deleted *Bcl-x* died, and thus the normal islets observed were derived from non-recombined, “escaper” β-cells that did not express the transgene-derived Cre. To assess this possibility, we isolated genomic DNA from pools of islets isolated from *RIP-Cre*; *Bcl-x^fl/fl^* mice and conducted PCR analysis with primers that would amplify and distinguish a recombined allele from a non-recombined allele (Figure 2B). Efficient recombination was detected in *RIP-Cre*; *Bcl-x^fl/fl^* islets. As expected, the recombined allele was not detected in controls lacking the RIP-Cre transgene (or in spleen from *RIP-Cre*; *Bcl-x^fl/fl^* mice, where Cre is not expressed).

### Assessing the functional role of Bcl-x in RIP1-Tag2 tumorigenesis

**Figure 3:**
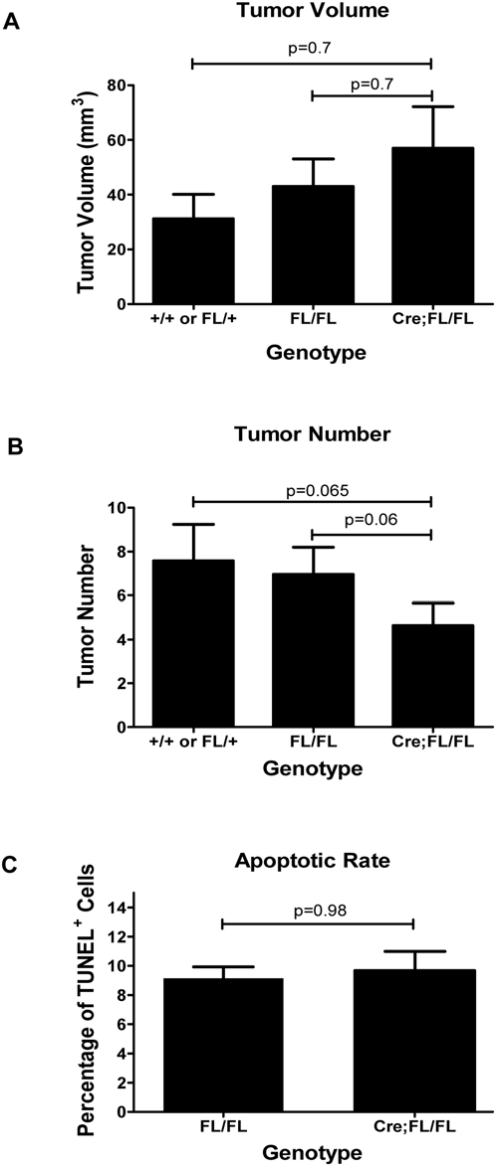
RIP1-Tag2 tumorigenesis is not dependent on Bcl-x function. (A and B) Comparison of tumor volume and number. Macroscopically visible tumors from 13 week old animals were excised, attached exocrine tissue removed and measured with ruler. Tumor volume was calculated using the formula V = (x * y^2^)×0.52. Data represent Mean±SEM; +/+ and FL/+: n = 7; FL/FL: n = 24; RIP-Cre;Fl/Fl: n = 22. Statistical significance was determined by non-parametric T-test (Mann-Whitney). P = 0.98 (D) Apoptotic rate. Apoptotic cells were quantified via TUNEL labeling of pancreatic sections from 13 week old animals. Data represent Mean±SEM (n = 5 mice/group). The percent TUNEL^+^ cells was calculated from sections of 16–17 individual tumors/group. Statistical significance determined by non-parametric T-test (Mann-Whitney). P = 0.98.

To investigate the role of Bcl-x in modulating apoptosis during RIP1-Tag2 tumorigenesis, we generated compound mice that were homozygous for the *Bcl-x^fl/fl^* allele and either carried both the RIP1-Tag2 and the RIP-Cre transgenes, or just the RIP1-Tag2 transgene. Additional controls included mice that carried the RIP1-Tag2 transgene, and either *Bcl-x^+/+^ or Bcl-x^fl/+^* alleles. We aged out cohorts of these mice to 13 weeks of age, and quantified both tumor burden (volume) and tumor number. The targeted knockout of *Bcl-x* did not result in a significant difference in tumor burden (Figure 3A). This essentially “wild-type” tumor burden in the knockout animals was accompanied by an approximately 40% decrease in tumor number; however, this effect did not reach statistical significance (Figure 3B). To determine if this lack of overt phenotypic effect on tumor growth was also reflected in the apoptotic rate, we performed TUNEL assays on formalin-fixed paraffin sections from *RIP1-Tag2*; *RIP-Cre*; *Bcl-x^fl/fl^* and *RIP1-Tag2*; *Bcl-x^fl/fl^* animals. As suggested by the tumor burden and tumor number data, there was no significant difference in the apoptotic rate in tumors from “knockout” versus control animals (Figure 3C).

**Figure 4:**
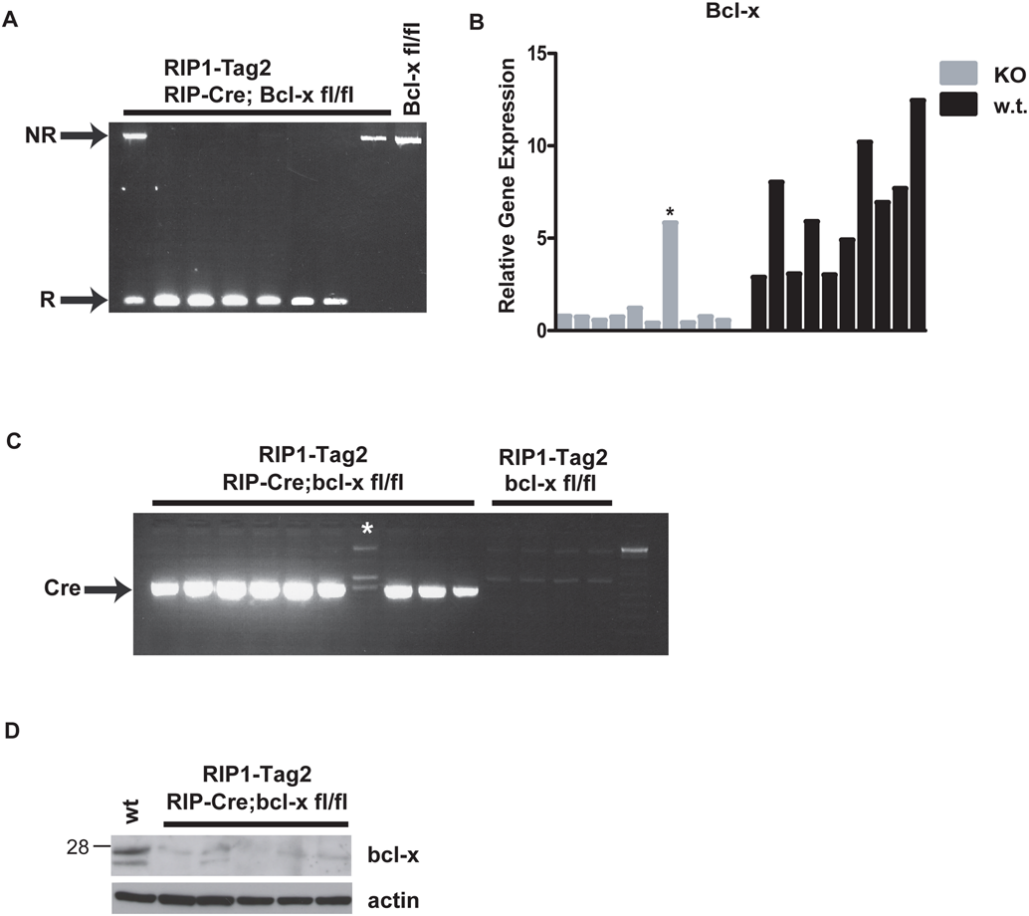
*Bcl-x* expression is ablated in the majority of *RIP1-Tag2*; *RIP-Cre*; *Bcl-x^fl/fl^* tumors. (A) Efficient recombination of *Bcl-x* in *RIP1-Tag2*; *RIP-Cre*; *Bcl-x^fl/fl^* tumors. Genomic DNA was isolated from individual tumors from *RIP1-Tag2*; *RIP-Cre*; *Bcl-x^fl/fl^* and *RIP1-Tag2*; *Bcl-x^fl/f^* ^*l*^ mice. PCR was carried out using a primer that binds upstream of the 5′ loxP site and another primer that binds 3′ to the downstream loxP site. The PCR fragment of the non-recombined allele and the recombined allele are 2.9 kb and 150 bp, respectively. (B) *Bcl-x* expression is significantly reduced in *Bclx*-KO tumors. Quantitative RT-PCR (Taqman) was carried out on 1^st^ strand cDNA synthesized from RNA isolated from individual tumors from *RIP1-Tag2*; *RIP-Cre*; *Bcl-x^fl/fl^* and *RIP1-Tag2*; *Bcl-x^fl/fl^*. A spanning probe that detected all major anti-apoptotic splice variants was used. Gene expression was normalized to and plotted as a function of GAPDH expression. (D) The RIP-Cre transgene is not expressed in *RIP1-Tag2*; *RIP-Cre*; *Bcl-x^fl/fl^* tumors that retain *Bcl-x* expression. Cre mRNA expression was monitored by RT-PCR on 1^st^ strand cDNA synthesized from RNA isolated from individual tumors from *RIP1-Tag2*; *RIP-Cre*; *Bcl-x^fl/fl^* and *RIP1-Tag2*; *Bcl-x^fl/fl^* mice. Following 27 cycles of PCR, the cDNA was fractionated on agarose gels and visualized by ethidium bromide staining. The PCR product is 520 bp. *non-recombined, Cre-negative tumor. (D) Bcl-x protein levels are significantly reduced in *Bcl-x*-KO tumors, as revealed by Western blotting as in Figure 1. β-actin is used as a loading control.

To verify that *Bcl-x* was efficiently deleted in the tumors arising in *RIP1-Tag2*; *RIP-Cre*; *Bcl-x^fl/fl^* mice, we monitored recombination using a PCR-based assay on genomic DNA from 12 randomly picked tumors (range 5.2 mm^3^–229 mm^3^): 10/12 tumors exhibited complete recombination, 1/12 showed partial recombination, and 1/12 had no recombination (Figure 4A and data not shown). Given the fact that the Cre transgene is not efficiently expressed in 10–20% of the β-cells, and noting that the ratio of tumors with recombined vs. non-recombined *Bcl-x* alleles was also ∼10%, we conclude that there was no selective growth advantage of *Bcl-x^+/+^* cells that manifested itself in the clonal outgrowth of β-cells harboring non-recombined alleles, in contrast to the situation with other genes that have critical roles in RIP1-Tag2 tumorigenesis (e.g. VEGF [26]). Notably, some of the largest tumors exhibited complete recombination, again supporting the notion that loss of Bcl-x function was not impairing tumor growth. As another measure of recombination efficiency, we assessed *Bcl-x* expression by quantitative RT-PCR (Taqman) on first strand cDNA derived from 10 independent *RIP1-Tag2*; *RIP-Cre*; *Bcl-x^fl/fl^* tumors and 10 *RIP1-Tag2*; *Bcl-x^fl/fl^* controls (Figures 4B and S1). There was a significant reduction in *Bcl-x* expression in 9/10 of the *RIP1-Tag2*; *RIP-Cre*; *Bcl-x^fl/fl^* tumors compared to *RIP1-Tag2*; *Bcl-x^fl/fl^* controls, indicating that *Bcl-x* was deleted in the majority of β-cells in these tumors. However, one tumor expressed wild-type levels of *Bcl-x*, suggesting that it arose from a β-cell that did not express Cre, and thus harbored a non-recombined allele. This prediction was confirmed by screening all 10 of the *RIP1-Tag2*; *RIP-Cre*; *Bcl-x^fl/fl^* tumors for Cre expression, and as predicted this exceptional tumor did not express Cre, in contrast to the 9 tumors that exhibited much reduced *Bcl-x* expression (Figure 4C). Excluding this Cre-negative, Bcl-x-positive “escaper” tumor from analysis, the mean *Bcl-x* expression in “KO” tumors was 9.5-fold lower than that of controls. Thus, deletion of *Bcl-x* in the β-cell compartment of these tumors resulted in a ∼90% reduction of *Bcl-x* expression, thereby confirming that the preponderance of *Bcl-x* expression in RIP1-Tag2 tumors is β-cell in origin and that Cre was efficiently deleting *Bcl-x* without apparent phenotypic consequence. The residual *Bcl-x* expression likely originates from the stromal component of the lesions (principally vascular and immune cell types), although we cannot formally rule out the possibility that these tumors are oligo-clonal, and that some of the residual *Bcl-x* mRNA is derived from relatively rare β-cells harboring non-recombined *Bcl-x* alleles. To determine whether the significant reduction of *Bcl-x* mRNA observed in bcl-x “KO” tumors resulted in a commensurate reduction in Bcl-x protein levels, we carried out a Western blot analysis on lysates from 5 independent *RIP1-Tag2;RIP-Cre*; *Bcl-x^fl/fl^* tumors. Consistent with the PCR-based analysis of recombination and the quantitative RT-PCR analysis of *Bcl-x* mRNA levels, Bcl-x protein levels were significantly reduced compared to wild-type (w.t.) tumors (Figure 4D), indicating Cre-mediated deletion of the *Bcl-x* locus in the vast majority of beta cells of *RIP1-Tag2;RIP-Cre*; *Bcl-x^fl/fl^* tumors. In addition, this result confirms that the immunoreactive bands detected in normal islets and RIP1-Tag2 lesions (Figure 1) were in fact derived from *Bcl-x*. Together, the data support the conclusion that Bcl-x does not have a central role in modulating tumor apoptosis in this pathway, and that its function is either non-essential, redundant, and/or readily compensated for by other genes, such as other anti-apoptotic members of Bcl-2 family.

### Expression profiling other Bcl-2 family members in islet tumorigenesis

**Figure 5:**
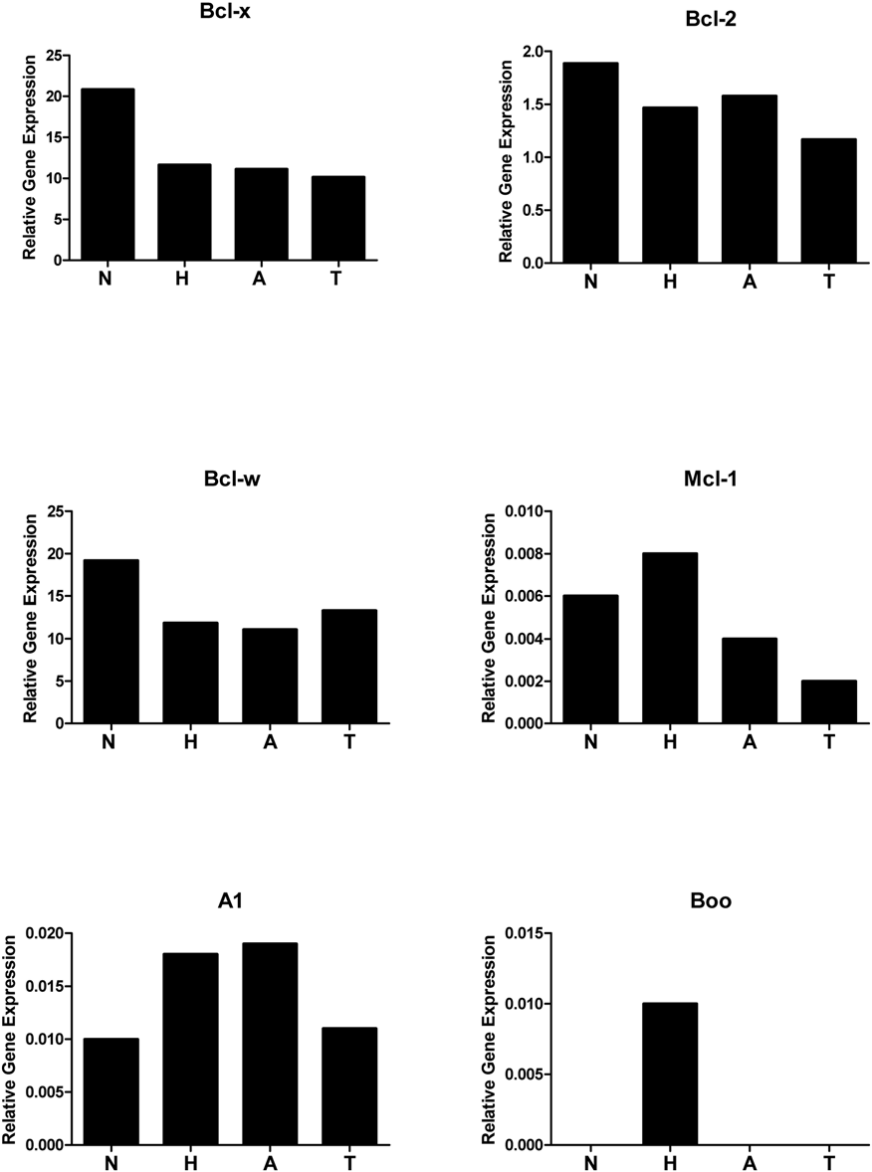
Expression of anti-apoptotic Bcl-2 family members in the stages of islet tumor development. The mRNA levels of the pro-survival Bcl-2 family members *Bcl-x*, *Bcl-2*, *Bcl-w*, *A1*, *Mcl-1* and *Boo* were assessed using quantitative RT-PCR (Taqman) on first strand cDNA synthesized from total RNA pools (4–6 animals per pool) of normal, non-transgenic islets, and of hyperplastic islets, angiogenic islets, and tumors from RIP1-Tag2 mice. Gene expression was normalized to and plotted as a percentage of GAPDH expression.

**Figure 6:**
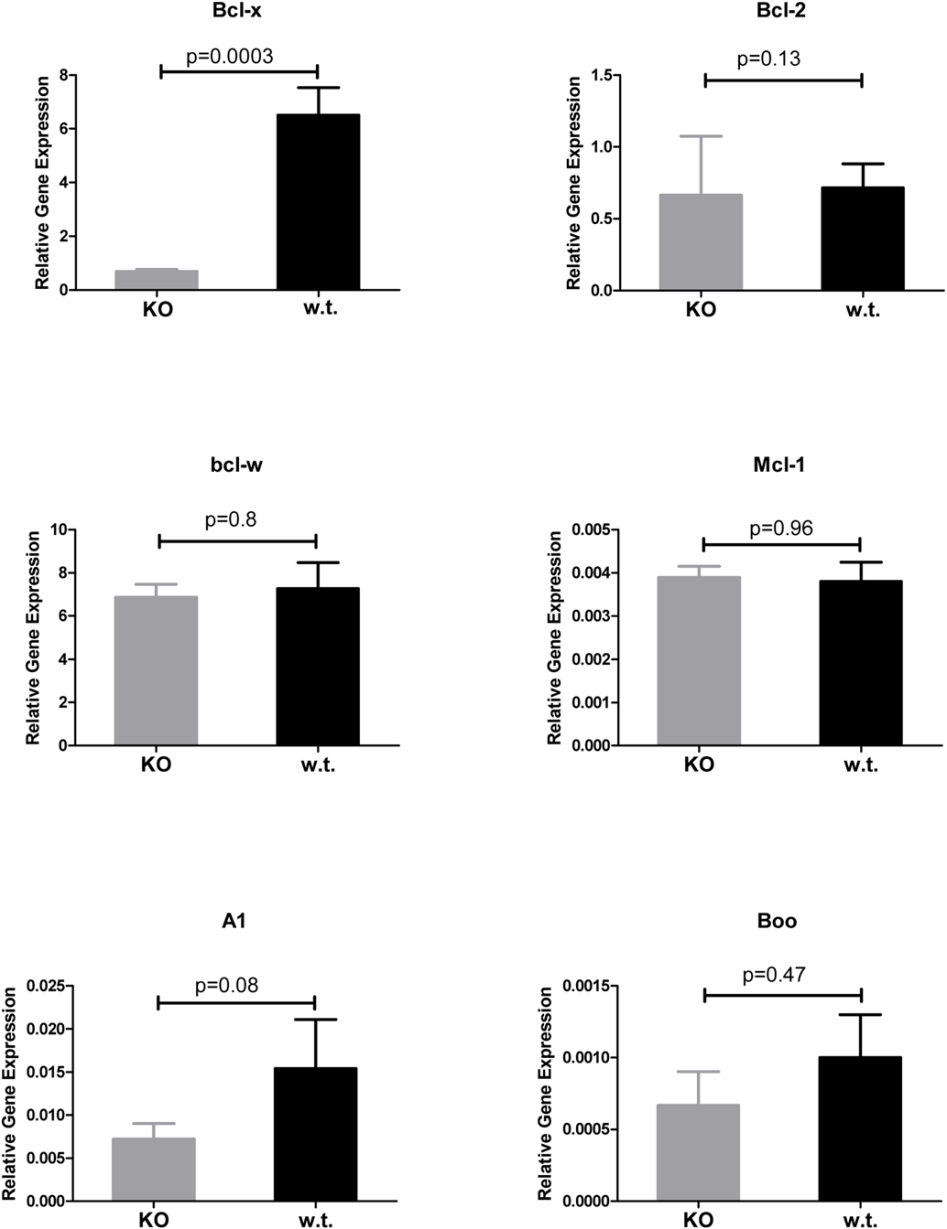
Loss of Bcl-x does not elicit transcriptional up-regulation of other anti-apoptotic Bcl-2 family members. The mRNA levels of *Bcl-x*, *Bcl-2*, *Bcl-w*, *A1*, *Mcl-1* and *Boo* were assessed using quantitative RT-PCR (Taqman) on first strand cDNA synthesized from total RNA isolated from individual tumors from *RIP1-Tag2*; *RIP-Cre*; *Bcl-x^fl/fl^* (n = 9 tumors from 5 mice; 1 tumor found to not express Cre and exhibit wild-type levels of *Bcl-x* excluded from these data) and *RIP1-Tag2*; *Bcl-x^fl/fl^* mice (n = 10 tumors from 5 individual mice). Relative gene expression was normalized to and presented as a percentage of GAPDH expression. Data represents mean±SEM. Statistical significance determined by non-parametric T-test (Mann-Whitney). P<0.05 considered significant.

Next, we sought to determine whether the 5 other known anti-apoptotic members of the Bcl-2 family might be expressed during RIP1-Tag2 tumorigenesis, thereby potentially compensating for the absence of Bcl-x. We quantified *Bcl-2*, *Bcl-w*, *A1*, *Mcl-1* and *Boo* mRNA levels using quantitative RT-PCR analysis (Taqman) on 1^st^ strand cDNA from normal islets (isolated from non-transgenic mice), hyperplastic islets, angiogenic islets, and tumors from RIP1-Tag2 mice. As shown in Figures 5 and S2, four of the genes were expressed; the fifth, *Boo*, was not detected in some of samples and was highly variable in replicate assays, consistent with its expression being largely restricted to the male and female reproductive organs [27]. Among the four expressed members, only *Bcl-w* was expressed at levels comparable to *Bcl-x*. Expression levels are presented relative to the control gene, GAPDH, which exhibited the least variable expression compared to L19 and Gus between normal islets and the different neoplastic islet lesions (Figure S3). To determine if one or more of the anti-apoptotic Bcl-2 family members was transcriptionaly up-regulated in the absence of Bcl-x and therefore potentially functionally compensating for the *Bcl-x* “knockout”, we performed quantitative RT-PCR analysis on the same 10 independent *RIP1-Tag2*; *RIP-Cre*; *Bcl-x^fl/fl^* and 10 *RIP1-Tag2*; *Bcl-x^fl/fl^* tumors in which *Bcl-x* expression was quantified (in Figure 4B). There proved to be no significant difference in the mRNA expression levels of these 5 genes (Figures 6 and S4). Additionally, Taqman analysis of the same tumor cDNAs for up-regulation of members of the IAP class of anti-apoptotic proteins (CIAP1, CIAP2, Survivin, XIAP, and BRUCE) similarly failed to reveal any significant transcriptional up-regulation of these genes in the *Bcl-x*-KO tumors (data not shown).

### Role of Bcl-x in modulating invasion in RIP1-Tag2 tumorignesis

**Figure 7:**
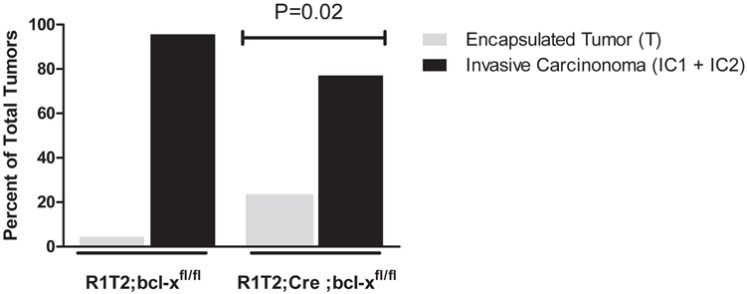
Loss of Bcl-x expression results in an altered proportion of adenomas and carcinomas. Tumors in H&E-stained sections from 13 wk *RIP1-Tag2*; *Bcl-x^fl/fl^* (n = 46 tumors from 5 mice) and *RIP1-Tag2*; *RIP-Cre*; *Bcl-x^fl/fl^* mice (n = 35 tumors from 5 mice) were scored as either non-invasive islet tumors/adenomas (IT) or invasive carcinomas (either IC1 or IC2) and the proportion of tumors in each class was calculated. Distribution of tumors types was compared between the two groups using Fisher's exact test. p = 0.017.

In addition to modulating apoptosis in tumor cells, there have been reports that Bcl-2 family members can additionally promote carcinogenesis via effects on tumor cell invasion [28]–[31]. In one report, a dissociation between the ability of Bcl-x_L_ to modulate apoptosis and invasiveness of human glioma cells was observed [29]. More recently, up-regulation of Bcl-x_L_ by somatic gene transfer during RIP1-Tag2 tumorigenesis was reported to increase invasiveness, without reducing apoptosis [32]. Motivated by these results, and by the evident lack of effect of loss of Bcl-x on overall RIP1-Tag2 tumor cell apoptosis and tumor burden, a potential role for Bcl-x in tumor invasion was assessed. Tumors from *RIP1-Tag2*; *Bcl-x^fl/fl^* and *RIP1-Tag2*; *RIP-Cre*; *Bcl-x^fl/fl^* mice were scored as non-invasive, encapsulated tumors (IT) or invasive carcinomas (IC1-microinvasive; IC2-highly invasive) according to the criteria of Lopez and Hanahan [16]. Interestingly, there was a significantly higher proportion of encapsulated, non-invasive tumors in *RIP1-Tag2*; *RIP-Cre*; *Bcl-x^fl/fl^* mice compared to *RIP1-Tag2*; *Bcl-x^fl/fl^* mice (22.8% vs. 4.3%; p<0.02) (Figures 7 and S5).

**Figure 8:**
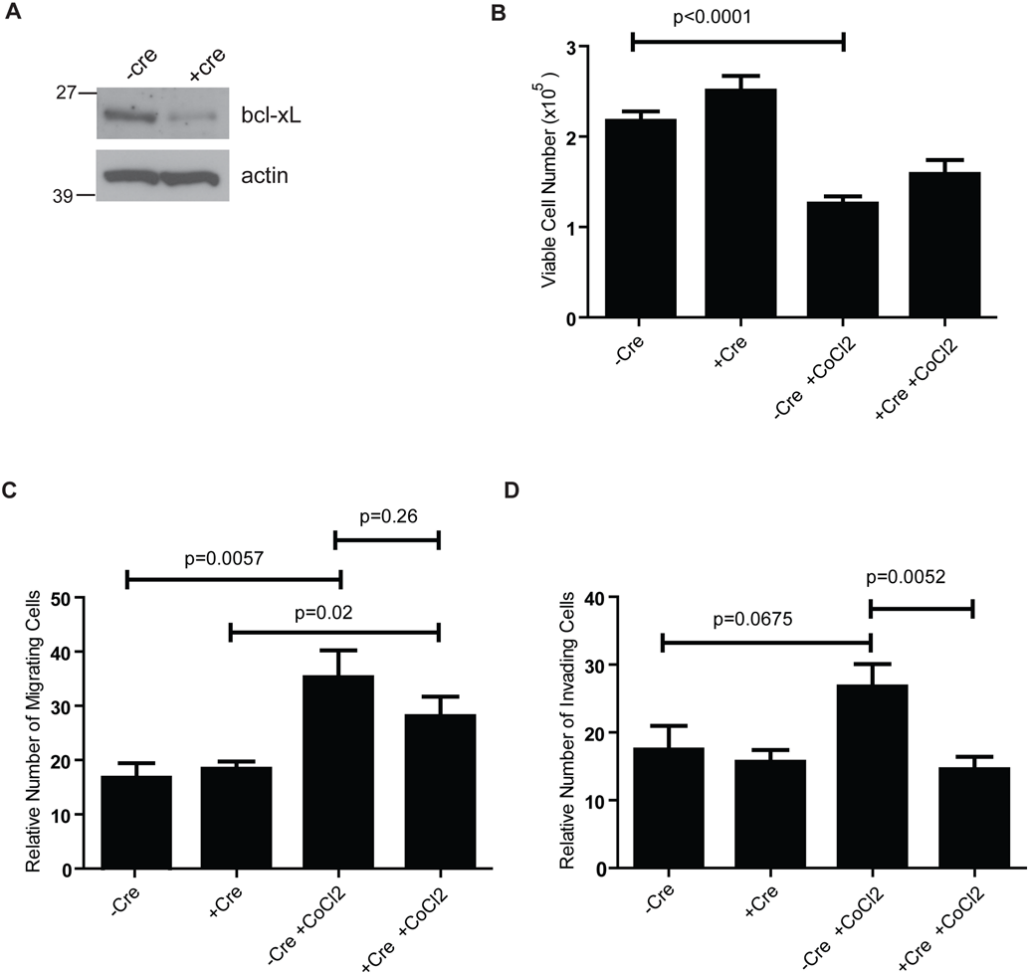
Effect of Bcl-x gene knockout on *in vitro* migration and invasion of tumor cells. βTC-*Bcl-x^fl/fl^* tumor cells were infected with adenovirus expressing Cre or control GFP and plated in triplicate in the presence or absence of cobalt chloride to assess the effect of reduced Bcl-x expression on cell migration and invasion. (A) Western blot of Bcl-x and actin protein levels in βTC-*Bcl-x* ^fl/fl^ cells treated +/−Cre at time of migration/invasion assays. (B) Cell growth. 2×10^5^ βTC-*Bcl-x^fl/fl^* cells +/−cre were plated in the presence or absence of CoCl_2_ and cells were counted after 48 hours. (C and D) Cell migration and invasion assays. Cells were plated in control trans-well inserts (C) or Matrigel-coated inserts (D). For the migration assay, results are plotted as the mean number of migrating cells/10× field (8 fields/membrane counted), while for the invasion assays the total number of invading cells/membrane are plotted. CoCl_2_ treatment resulted in a significant decrease in invasion of βTC-Bcl-x-KO cells compared to wild-type cells (P = 0.005). Results shown represent the mean±SEM of results from three independent experiments performed in triplicate and are corrected for changes in total cell number (from B) compared to control cells (-Cre, -CoCl_2_).

To substantiate the effect of loss of Bcl-x on invasion, cancer cells with wild-type or deleted *Bcl-x* (βTC-Bcl-x-wt or βTC-Bcl-x-knockout [KO]) were generated by deriving β-tumor cell lines (“βTC”) from tumors of *RIP1-Tag2*; *Bcl-x^fl/fl^* mice, and infecting the cells with adenoviruses that do or do not express Cre (Ad-Cre-GFP or Ad-GFP). Two weeks post-infection with Ad-Cre-GFP, the levels of Bcl-x protein within the cell population were reduced by approximately 75–80% (Figure 8A), likely reflecting recombination of the *Bcl-x* gene in the majority of cells; serum-starved cells +/−Cre were seeded into the upper chambers of porous transwell inserts either uncoated or Matrigel-coated to assess the effect of loss of *Bcl-x* expression on migration and invasion, respectively. To account for any potential differences in cell growth between βTC-Bcl-x-wt or βTC-Bcl-x-KO cells over the time course of the migration/invasion assays, equal numbers of cells were plated in triplicate, and total cell numbers determined after a 48-hour incubation. During this time period, there was no apparent difference in cell growth between wild-type and Bcl-x-KO cells (Figure 8B). This was consistent with the lack of effect of loss of Bcl-x function on tumor growth and apoptosis in vivo. In addition, there was no significant difference in the number of migrating or invading cells along a serum gradient (Figure 8C,D). However, the βTC cells were poorly invasive in these ex vivo assays, as reported previously [32], in contrast to the common appearance of invasive cancer cells during tumor progression. We wondered, therefore, whether the typical cell culture conditions might not properly model the invasion-inducing microenvironment in vivo. Notably, various reports, including previous studies in the RIP1-Tag2 model, have demonstrated a role for hypoxia in stimulating tumor invasion [33]–[36]. We therefore repeated the migration/invasion assays in conditions mimicking hypoxia, by incorporating cobalt chloride (CoCl_2_), which has been shown to induce biochemical responses similar to those observed under low oxygen conditions [37], [38]. In this condition, overall cell survival was reduced (Figure 8B) though the wild-type cells evidenced a 2-fold increase in the number of migrating cells (p = 0.006), and a 1.5-fold increase in invading cells (p = 0.07) (Figure 8C,D) relative to the total number of surviving cells. A similar increase in migration was observed in response to CoCl_2_ treatment of the βTC-Bcl-x-KO cells (p = 0.02) (Figure 8C); in contrast, these cells did not exhibit increased invasion under these pseudo-hypoxic conditions, and compared to the wild-type cells exhibited a modest yet significant decrease (45%; p = 0.005) in the relative number of invading cells (Figure 8D). These data, consistent with the in vivo analysis, suggest that while expression of multiple Bcl-2 family members may render Bcl-x functionally redundant in terms of effects on tumor cell apoptosis, Bcl-x may exhibit non-redundant and distinct functions in regard to tumor invasiveness.

## Discussion

In this study, we sought to determine whether genetic deletion of one prominent anti-apoptotic member of the Bcl-2 family, Bcl-x, would impact tumorigenesis. We demonstrate that Bcl-x is non-essential for RIP1-Tag2 tumorigenesis, and that cells harboring null alleles of *Bcl-x* do not have an inherent survival disadvantage to cells expressing wild type Bcl-x. In addition, we demonstrate that 4 of the other anti-apoptotic Bcl-2 family members, *Bcl-2*, *Bcl-w*, *A1* and *Mcl-1* are reproducibly expressed in normal, non-transgenic islets and throughout multistage tumorigenesis in RIP1-Tag2 mice. Importantly, in tumors in which *Bcl-x* is deleted, mRNA expression of these genes remains unchanged, suggesting that Bcl-x in this tumor pathway is either non-essential, or functionally redundant, and thus readily substituted without overt transcriptional up-regulation of other members of this gene class. Based on the quantitative RT-PCR experiments presented in this study, a potential source of functional redundancy is Bcl-w, which appears to be expressed at significantly higher levels than Bcl-2, A1, Mcl-1 and Boo in both normal islets and lesional stages of the RIP1-Tag2 tumor pathway.

Our results are consistent with a recent study demonstrating that Bcl-2 is not strictly required for the development of *myc* induced lymphomas in Eu-myc transgenic mice [39]. Whereas previous studies had shown that *myc* plus over-expression of *Bcl-2* synergized to accelerate lymphomagenesis in the Eu-myc model [40], the genetic ablation of *Bcl-2* had no impact on the cancer phenotype. Similarly, in contrast to the loss of function studies presented above, forced over-expression of *bcl-x_L_* via a RIP7-*Bcl-x_L_* transgene has a profound effect on RIP1-Tag2 tumorigenesis, in that tumor progression is accelerated and tumor burden increased in the double transgenic mice [15]. This augmentation of tumorigenesis is accompanied by a significant decrease in the apoptotic rate. Similar results have been obtained in a model of islet cell tumorigenesis driven by an inducible c-myc oncogene, wherein upregulation of *bcl-x_L_* is obligatory for *myc*-induced islet tumorigenesis [20]. These data indicate that *bcl-x_L_* is a potent anti-apoptotic gene and tumor progression factor when up-regulated/over-expressed during lymphoid and pancreatic islet tumorigenesis, but that its anti-apoptotic functions are in some circumstances non-essential, likely due to the redundant functions of the other expressed Bcl-2 family members.

Despite the evident redundancy of endogenous levels of Bcl-x in impacting apoptosis and tumor growth, a potentially distinct role of Bcl-x in influencing tumor invasion was revealed by the observation of an increased proportion of non-invasive adenomas compared to invasive carcinomas in mice lacking Bcl-x expression in the oncogene-expressing islet β-cells. This histopathological implication is supported by experiments with islet tumor cell lines lacking Bcl-x expression. The Bcl-x-null cells exhibited a decreased capability to invade under conditions mimicking hypoxia; in contrast, when the same cells were assayed in normal conditions, their invasiveness was comparable to Bcl-x proficient cancer cells. This result suggests that while endogenous Bcl-x expression may be redundant under normal conditions, it can play a distinct role in stressed conditions (e.g. hypoxia, nutrient deprivation, chemotherapy).

Our findings implicating Bcl-x in invasion are notable in light of a recent study by Du et al., who reported a significant increase in the incidence of invasive carcinomas in RIP1-Tag2 mice engineered to focally over-express Bcl-x_L_ via retrovirus-mediated somatic gene transfer into neoplastic lesions [32]. Interestingly, this mode of up-regulating Bcl-x_L_ did not affect the incidence of apoptotic cells in the tumors, in contradistinction to the aforementioned study in which the RIP-Bcl-x_L_ transgene produced a decreased apoptotic index [15]; such phenotypic differences may be explained by differences in the uniformity, timing or levels of expression of Bcl-x_L_ (the effect of the transgene-mediated over-expression of Bcl-x_L_ on tumor invasion was not assessed in this earlier study). Our current investigation now provides additional, complimentary data to that of Du et al in regard to this emergent function for Bcl-x, by demonstrating that endogenous Bcl-x, in the absence of overt over-expression, can influence tumor invasion.

Therapeutics that engage programmed cell death in tumor cells have promise for the treatment of cancer. Indeed compounds that broadly fall into this class have entered clinical development. ABT-737, a BH3 mimetic, is one example [41]. Recently, it has been reported that ABT-737 is not cytotoxic to all tumors cells, and that “chemoresistance” to ABT-737 is dependent on appreciable levels of Mcl-1 expression, the one Bcl-2 family member it does not effectively inhibit [42], [43]. Indeed, suppression of Mcl-1 protein levels by shRNA knockdown or indirect pharmacological downregulation sensitizes resistant tumor cells to ABT-737 mediated cell death [44], [45]. Collectively, the data suggest that activity among the pro-survival Bcl-2 family members is, in certain settings, functionally redundant, and that tumor cells utilize multiple Bcl-2 family members for survival and proliferation. The prediction follows that treatment of the RIP1-Tag2 mice with pan-Bcl inhibitors will disrupt tumor growth if indeed, (as expected) these genes cooperate in modulating the propensity for apoptosis as an intrinsic barrier to tumorigenesis. It will be of additional interest to ascertain whether such Bcl inhibitors can also restrict tumor invasion and malignancy, endpoints that could be of relevance in the clinical setting.

## Materials and Methods

### Real-time Q-PCR Analysis

Quantitative PCR was performed essentially as in Elson et al.[46] In brief, RNA was extracted using Qiagen RNase Easy as per manufacturer's instructions. RNA concentration was determined by spectrophotometer (NanoDrop, Rockland, DE). RNA quality was confirmed using a Bioanalyser (Agilent Technologies, Palo Alto, CA). Total RNA was incubated with DNase (DNA-free, Ambion, Austin, TX) to remove contaminating DNA. The DNase was inactivated and removed according to the manufacturer's specifications. ‘No Reverse transcriptase’ controls were performed on all samples to confirm that genomic DNA was not present. RNA was reverse transcribed into cDNA with iScript (Bio-Rad, Hercules CA), 300 ng in a 20 ul volume according to manufacturer's specifications. Quantitative-PCR analysis was performed on an AB Prism 7900 or 7700 sequence detection system (Applied Biosystems, Foster City, CA). Quantitative detection of specific nucleotide sequences was based on the fluorogenic 5′ nuclease assay, and relative expression was calculated as previously described [46]. Assays were designed (using Primer Express software v1.5, Applied Biosystems) with 6-FAM fluorophore on the 5′ end and the quencher BHQ1 on the 3′ end and reactions were optimized to have >90% efficiency [or purchased as Assays–on-Demand from Applied Biosystems(AB)]. Primer and probe concentrations of 500 nM and 200 nM, were used respectively. The cDNA equivalent to 3–5 ng of RNA was measured in triplicate by real time PCR using QPCR master mix with final concentrations 5.5 mM MgCl2, 200 uM dNTPs and 0.5units Hotstart Amplitaq Gold (AB) in 20 ul volume 384 well plate or 50 uL volume for 96 well plates. For normalization, cDNA equivalent to 3–5 ng input RNA was measured for GAPDH, L19 and Gus. GAPDH expression was the least variable under these experimental conditions (Figure S3).

For the detection of transcriptional target messages, the following primer and probe sets were used: pan-*Bcl-x*: ABI Assay on Demand (AOD) Mm00437783_m1; *Bcl-w*: AOD mM00432054_m1; *Bcl-2*: AOD Mm00477631_m1; *Mcl-1*: AOD Mm00725832_s1; *A1*: AOD Mm00833201_s1; *Boo*: AOD; Mm00478988_m1.

### Transgenic mice breeding

The generation of RIP1-Tag2 mice as a model of pancreatic islet cell carcinogenesis has been previously reported [13]. RIP2-Cre transgenic mice, kindly provided by Mark Magnuson (Vanderbilt University Medical Center, Nashville, TN) [23], were previously backcrossed into the C57-Bl/6J background for more than 15 generations. *Bcl-x*-floxed allele animals (mixed C57BL/6–129SvEv background) were previously described [25], [47]. They were backcrossed into the C57-Bl/6J background for 5 generations, then crossed with the RIP-Tag2 and the RIP2-Cre in a *Bcl-x^fl/wt^* heterozygous background and then intercrossed to *Bcl-x^fl/fl^* animals to obtain *RIP-Tag2*; *RIP2-Cre*; *Bclx^fl/fl^* animals. All experiments involving mice were approved by the University of California, San Francisco (UCSF) institutional review board.

### Tissue preparation and immunohistochemistry

Normal (from non-transgenic C57Bl/6 mice), hyperplastic islets (from 4–6 wk RIP1-Tag2 mice; not red), and angiogenic islets (from 8–10 wk RIP1-Tag2 mice; red, with evident hemorrhages) were isolated as previously described [48]. Tumors were carefully microdissected away from the surrounding exocrine pancreas tissue of freshly excised pancreata (from 12–13 wk RIP1-Tag2 mice). For protein analysis, tissue was disrupted in RIPA buffer containing a protease inhibitor cocktail (Roche); western blotting was performed using antibodies to Bcl-x (Pharmingen) and β-actin (Sigma) for a loading control. For immunohistochemical analysis, mice were anesthetized with tribromoethanol (Sigma) and heart-perfused with PBS and then 10% zinc-buffered formalin (Medical Chemical Corp.). Pancreata were removed and post-fixed in 10% zinc-buffered formalin overnight and processed for paraffin embedding as previously described [16]. TUNEL staining and H&E grading were performed as previously described [16], [26]. TUNEL quantification was performed on tumor-containing sections (5 µm paraffin) from at least 5 mice per group. Statistical analysis was performed using the non-parametric t-test (Mann-Whitney) and p values of less than 0.05 were considered statistically significant. For tumor grading, paraffin blocks from pancreases of 5 mice/group were sectioned and 5 slides 50 µm apart were stained by H&E. Tumors were blindly graded as either encapsulated, non-invasive tumors (IT) or invasive carcinoma type 1 or 2 (IC1,2) as described [16]. Differences in the proportion of IT and IC tumors in the two different genotypes were assessed by Fisher's exact test and a p value of less than 0.05 was considered statistically significant.

### Determination of tumor burden

Quantification of tumor burden was determined as previously described [49]. Briefly, tumors were microdissected and tumor dimensions were measured with a ruler, and tumor volume was calculated using the formula [volume = width^2^×length×0.52] to approximate the volume of spheroid. Tumor burden per mouse was calculated as the sum of the volumes of all tumors per mouse. Statistical significance was determined by non-parametric T-test (Mann-Whitney) and p values of less than 0.05 were considered statistically significant.

### In vitro migration and invasion assays

A βTC *bcl-x^fl/fl^* cell line (#14731) was derived from a tumor of a *RIP1-Tag2*; *Bcl-x^fl/fl^* mouse. Briefly, the dissected tumor was disrupted in DMEM supplemented with 20% fetal bovine serum (FBS), penicillin/streptomycin, and fungizone and tumor cells were collected by gravity sedimentation. Cells were infected with either Ad-GFP or Ad-Cre-GFP (Vector Development Lab, Baylor College of Medicine) at a multiplicity of infection of ∼200. Approximately two weeks post-infection, cells were serum-starved overnight and 1×10^5^ cells were seeded the next day in triplicate into BD BioCoat control trans-well inserts with 8-µm porous polyethelene terepththalate (PET) membranes (for migration assays) or Matrigel-coated invasion chambers (BD Biosciences). Cells were plated in serum-free DMEM containing 0.2% bovine serum albumin +/−100 µM cobalt chloride into the upper chambers and the lower chambers were filled with DMEM (+/−100 µM CoCl_2_) containing 10% FBS as a chemoattractant. Cells were incubated for 48 hours in a humidified 5% CO_2_/37°C incubator after which non-migrated/invaded cells were removed from the upper membrane and membranes were fixed and stained with 0.2% crystal violet. For quantitating migration, the mean number of migrating cells in eight, 10× fields was determined/membrane; for quantitating invasion, the total number of invading cells/membrane was counted (under 10× objective) due to the low number of invading cells. To determine whether differences in cell growth/survival as a result of reduced Bcl-x expression and/or CoCl_2_ treatment could be contributing to the migration/invasion results, in parallel with plating cells for the migration/invasion assays, 2×10^5^ βTC *Bcl-x^fl/fl^* cells +/−Ad-Cre were plated in triplicate in 24-well plates (+/−CoCl_2_) and cells were trypsinized and counted after 48 hrs. The migration/invasion and cell growth assays were repeated in three separate experiments. With each experiment, the same βTC *Bcl-x^fl/fl^* cells +/−cre were also plated for generation of protein extracts to confirm reduced Bcl-x protein expression.

## Supporting Information

Figure S1 Bcl-x expression is significantly reduced in Bclx-KO tumors. Quantitative RT-PCR (Taqman) was carried out on 1st strand cDNA synthesized from RNA isolated from individual tumors from RIP1-Tag2; RIP-Cre; Bcl-xfl/fl and RIP1-Tag2; Bcl-xfl/fl mice. A pan probe that detected all major anti-apoptotic splice variants was used. Gene expression was normalized to and plotted as a function of GUS (A) or L19 expression (B).
(0.13 MB TIF)

Figure S2 Expression of anti-apoptotic Bcl-2 family members during RIP1-Tag2 tumor development. mRNA levels of individual pro-survival Bcl-2 family members, Bcl-w, A1, Mcl-1 and Boo were assessed using quantitative RT-PCR (Taqman) on first strand cDNA synthesized from total RNA pools (4–6 animals per pool) of normal, non-transgenic islets, hyperplastic islets, angiogenic islets, and tumors from RIP1-Tag2 mice. Gene expression was normalized to and plotted as a percentage of GUS (A) or L19 expression (B).
(0.22 MB TIF)

Figure S3 Control Gene expression during RIP1-Tag2 tumor progression and in Bclx-w.t. and KO tumors. (A–C) mRNA levels were assessed using quantitative RT-PCR (Taqman) on first strand cDNA synthesized from total RNA pools (4–6 animals per pool) of normal, non-transgenic islets (N), hyperplastic islets (H), angiogenic islets (A), and tumors (T) from RIP1-Tag2 mice. Three distinct Taqman primer sets were used to detect GAPDH, GUS and L19. (D) Quantitative RT-PCR (Taqman) was carried out on 1st strand cDNA synthesized from RNA isolated from individual tumors from RIP1-Tag2; RIP-Cre; Bcl-xfl/fl and RIP1-Tag2; Bcl-xfl/fl mice.
(0.18 MB TIF)

Figure S4 Loss of Bcl-x does not elicit transcriptional up-regulation of other anti-apoptotic Bcl-2 family members. mRNA levels of individual pro-survival Bcl-2 family members, Bcl-x, Bcl-2, Bcl-w, A1, Mcl-1 and Boo were assessed using quantitative RT-PCR (Taqman) on first strand cDNA synthesized from total RNA isolated from individual tumors from RIP1-Tag2; RIP-Cre; Bcl-xfl/fl mice (n = 9 tumors from 5 distinct mice; 1 tumor found to not express Cre and exhibit wild-type levels of Bcl-x excluded from these data) and RIP1-Tag2; Bcl-xfl/fl mice (n = 10 tumors from 5 individual mice). Relative gene expression was normalized to and presented as a percentage of GUS (A) or L19 (B) expression. The apparent up-regulation of bcl-w and mcl-1 when normalized to L19 expression does not represent an actual change in Bcl-2 family member expression but rather lower level of L19 in KO tumors (Figure S3). Data represents Mean±SEM. Statistical significance determined by non-parametric T-test (Mann-Whitney). P<0.05 considered significant.
(0.20 MB TIF)

Figure S5 Loss of Bcl-x expression results in an altered proportion of adenomas/carcinomas. Tumors on H&E sections from 13 wk RIP1-Tag2; Bcl-xfl/fl (n = 46 tumors from 5 mice) and RIP1-Tag2; RIP-Cre; Bcl-xfl/fl mice (n = 35 tumors from 5 mice) were scored as either non-invasive islet tumors/adenomas (IT), micro-invasive carcinomas (type 1; IC1), or highly invasive carcinomas (type 2; IC2) and the proportion of tumors in each class was calculated. p = 0.04, Chi-squared test for independence, comparing distribution of tumor types between the two groups of mice.
(0.12 MB TIF)
