# Supplementary figures and images for: Genetic Ablation of Bcl-x Attenuates Invasiveness without Affecting Apoptosis or Tumor Growth in a Mouse Model of Pancreatic Neuroendocrine Cancer
Source: PLoS One. 2009 Feb 11;4(2):e4455. doi: 10.1371/journal.pone.0004455 (PMC2635964; doi:10.1371/journal.pone.0004455)

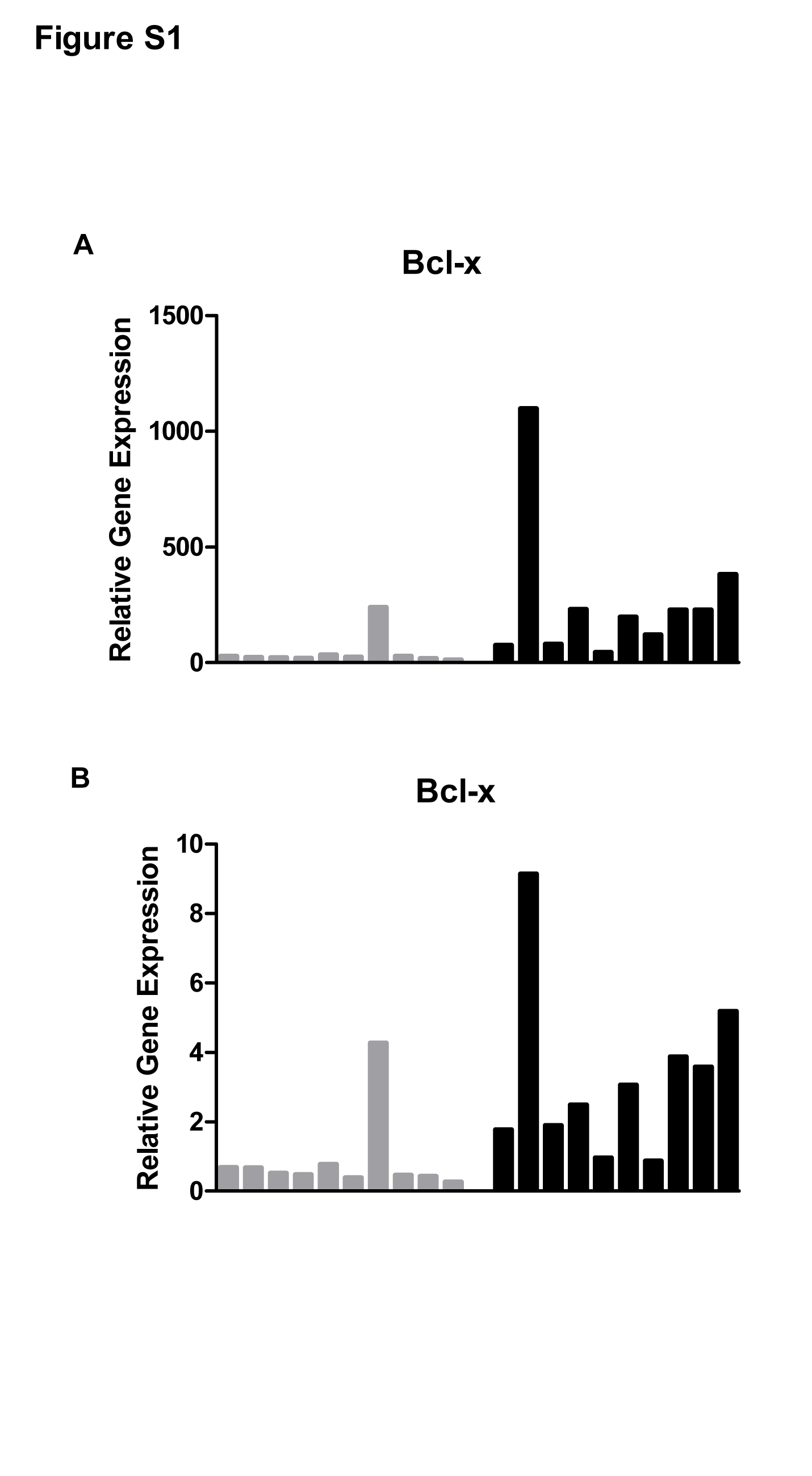

Supplement: Figure S1 — Bcl-x expression is significantly reduced in Bclx-KO tumors. Quantitative RT-PCR (Taqman) was carried out on 1st strand cDNA synthesized from RNA isolated from individual tumors from RIP1-Tag2; RIP-Cre; Bcl-xfl/fl and RIP1-Tag2; Bcl-xfl/fl mice. A pan probe that detected all major anti-apoptotic splice variants was used. Gene expression was normalized to and plotted as a function of GUS (A) or L19 expression (B). (0.13 MB TIF) [file pone.0004455.s001.tif]

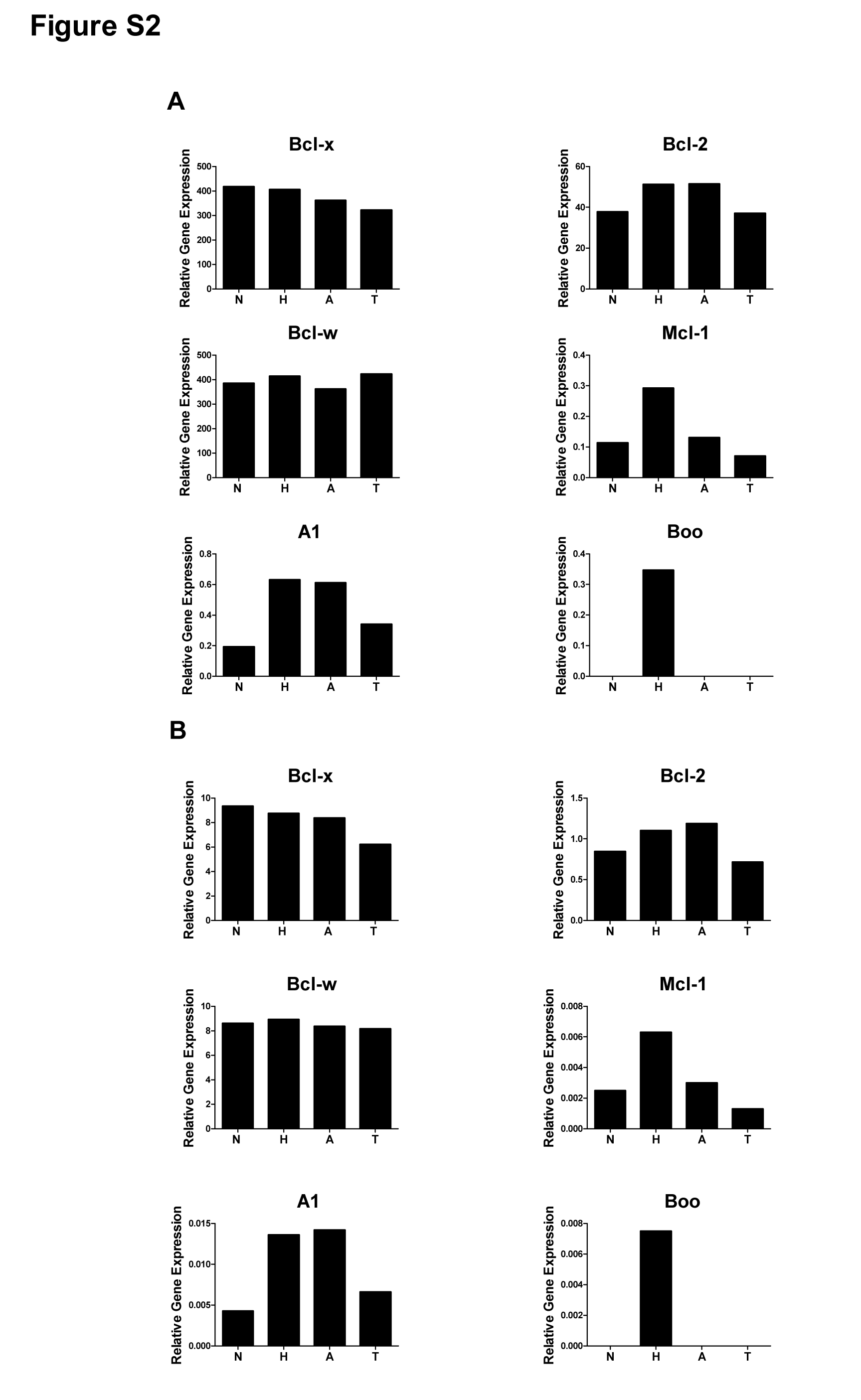

Supplement: Figure S2 — Expression of anti-apoptotic Bcl-2 family members during RIP1-Tag2 tumor development. mRNA levels of individual pro-survival Bcl-2 family members, Bcl-w, A1, Mcl-1 and Boo were assessed using quantitative RT-PCR (Taqman) on first strand cDNA synthesized from total RNA pools (4–6 animals per pool) of normal, non-transgenic islets, hyperplastic islets, angiogenic islets, and tumors from RIP1-Tag2 mice. Gene expression was normalized to and plotted as a percentage of GUS (A) or L19 expression (B). (0.22 MB TIF) [file pone.0004455.s002.tif]

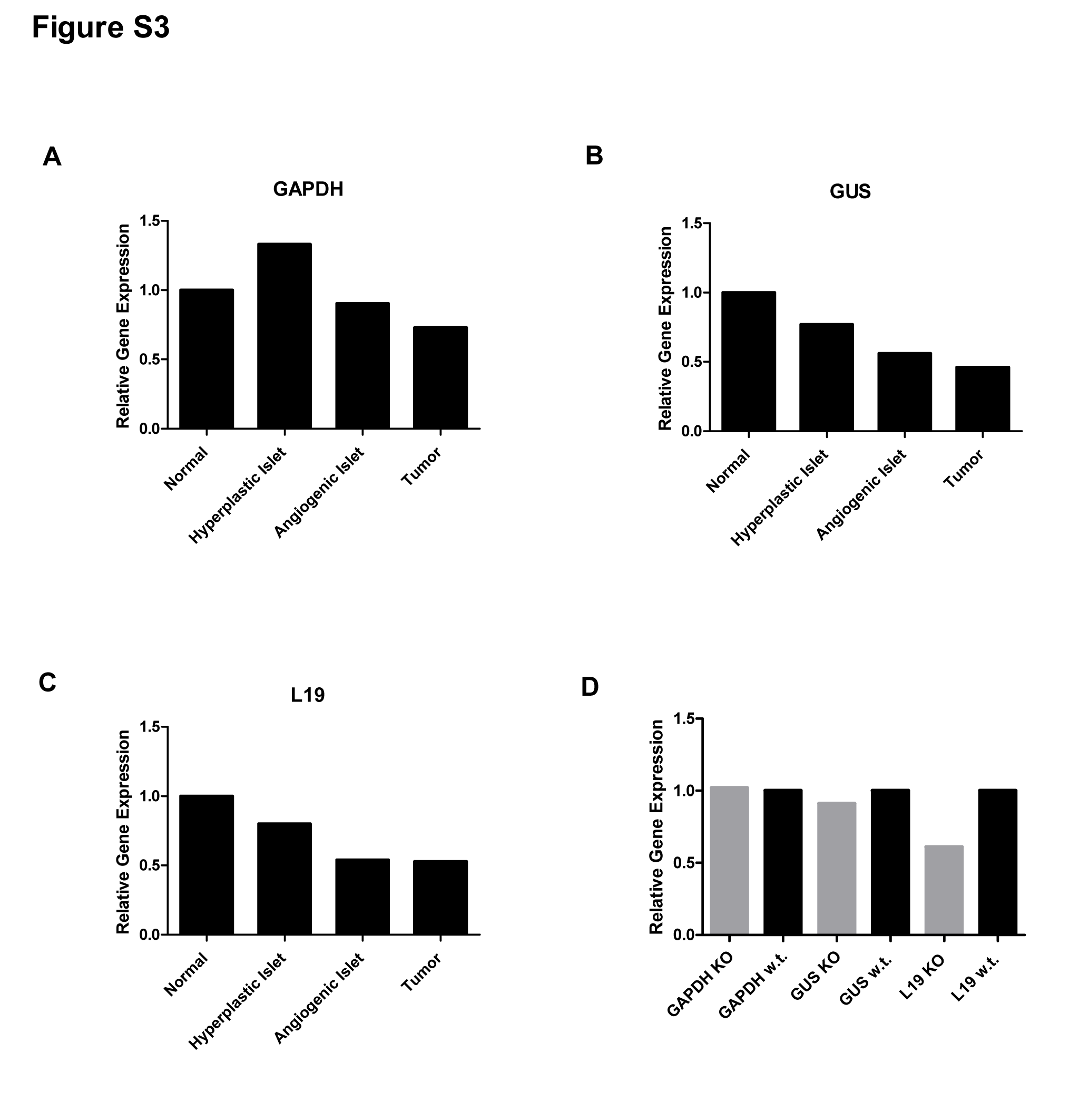

Supplement: Figure S3 — Control Gene expression during RIP1-Tag2 tumor progression and in Bclx-w.t. and KO tumors. (A–C) mRNA levels were assessed using quantitative RT-PCR (Taqman) on first strand cDNA synthesized from total RNA pools (4–6 animals per pool) of normal, non-transgenic islets (N), hyperplastic islets (H), angiogenic islets (A), and tumors (T) from RIP1-Tag2 mice. Three distinct Taqman primer sets were used to detect GAPDH, GUS and L19. (D) Quantitative RT-PCR (Taqman) was carried out on 1st strand cDNA synthesized from RNA isolated from individual tumors from RIP1-Tag2; RIP-Cre; Bcl-xfl/fl and RIP1-Tag2; Bcl-xfl/fl mice. (0.18 MB TIF) [file pone.0004455.s003.tif]

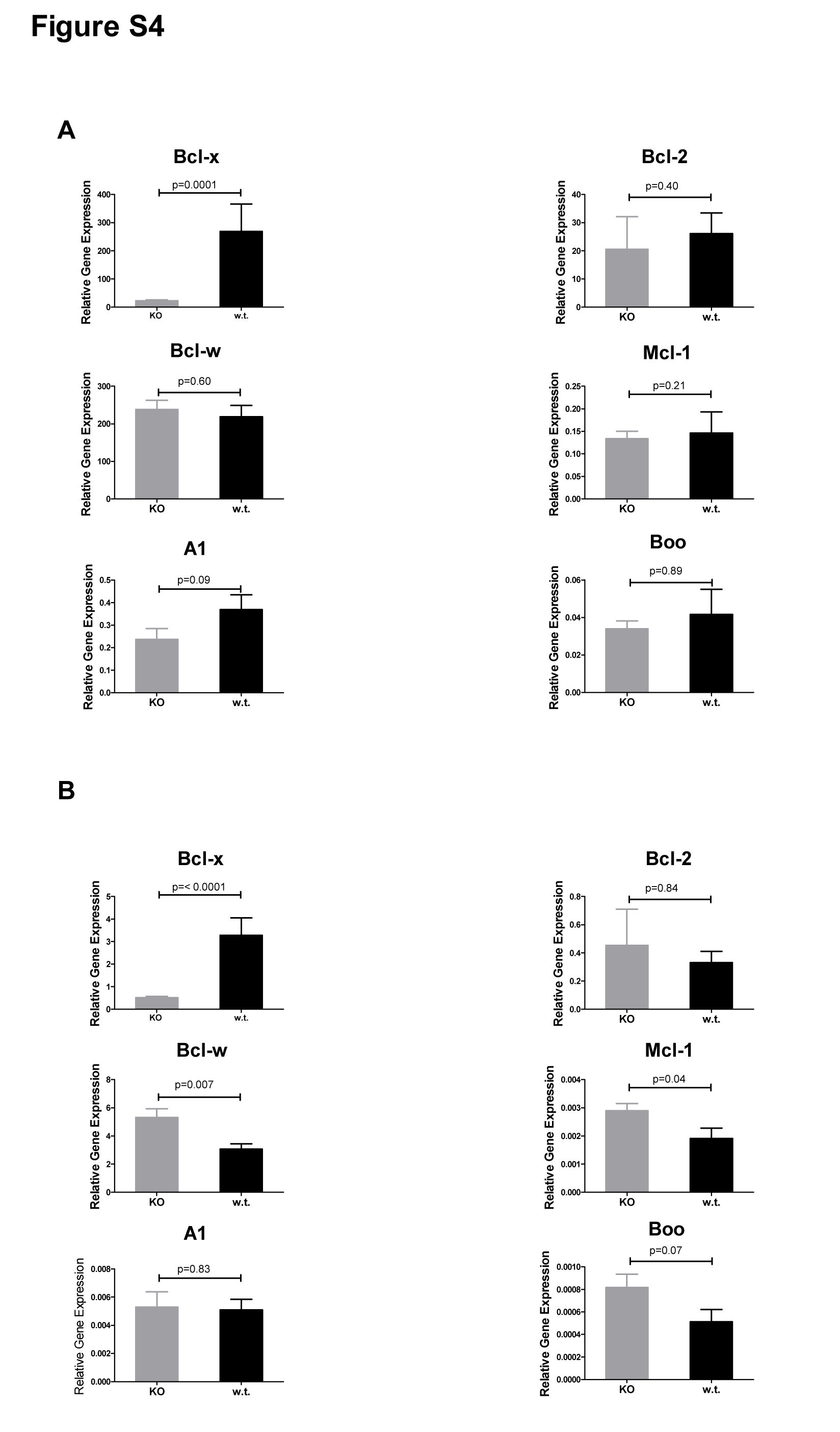

Supplement: Figure S4 — Loss of Bcl-x does not elicit transcriptional up-regulation of other anti-apoptotic Bcl-2 family members. mRNA levels of individual pro-survival Bcl-2 family members, Bcl-x, Bcl-2, Bcl-w, A1, Mcl-1 and Boo were assessed using quantitative RT-PCR (Taqman) on first strand cDNA synthesized from total RNA isolated from individual tumors from RIP1-Tag2; RIP-Cre; Bcl-xfl/fl mice (n = 9 tumors from 5 distinct mice; 1 tumor found to not express Cre and exhibit wild-type levels of Bcl-x excluded from these data) and RIP1-Tag2; Bcl-xfl/fl mice (n = 10 tumors from 5 individual mice). Relative gene expression was normalized to and presented as a percentage of GUS (A) or L19 (B) expression. The apparent up-regulation of bcl-w and mcl-1 when normalized to L19 expression does not represent an actual change in Bcl-2 family member expression but rather lower level of L19 in KO tumors (Figure S3). Data represents Mean±SEM. Statistical significance determined by non-parametric T-test (Mann-Whitney). P<0.05 considered significant. (0.20 MB TIF) [file pone.0004455.s004.tif]

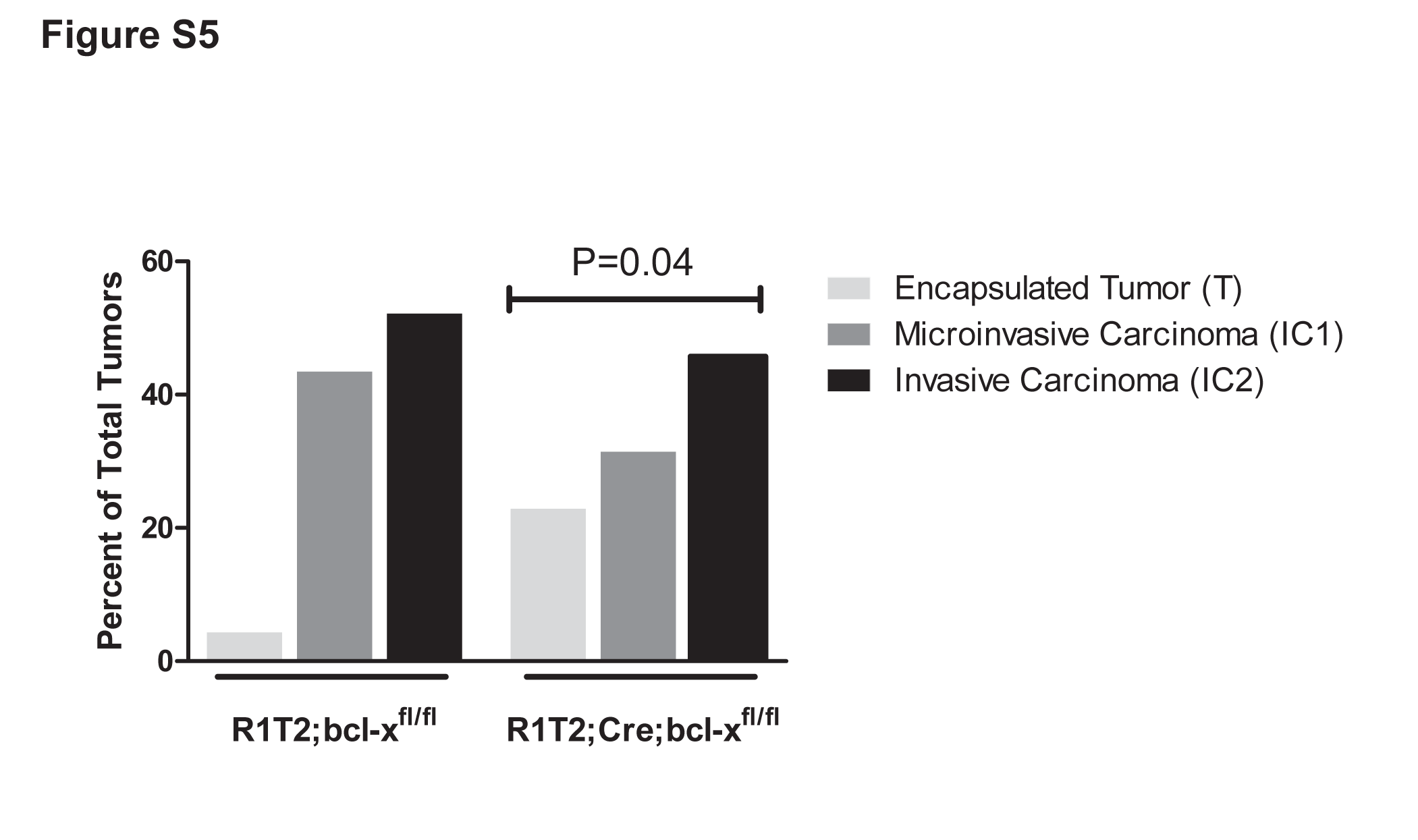

Supplement: Figure S5 — Loss of Bcl-x expression results in an altered proportion of adenomas/carcinomas. Tumors on H&E sections from 13 wk RIP1-Tag2; Bcl-xfl/fl (n = 46 tumors from 5 mice) and RIP1-Tag2; RIP-Cre; Bcl-xfl/fl mice (n = 35 tumors from 5 mice) were scored as either non-invasive islet tumors/adenomas (IT), micro-invasive carcinomas (type 1; IC1), or highly invasive carcinomas (type 2; IC2) and the proportion of tumors in each class was calculated. p = 0.04, Chi-squared test for independence, comparing distribution of tumor types between the two groups of mice. (0.12 MB TIF) [file pone.0004455.s005.tif]
